# Report of Laparotomy for Obstruction of Bowels

**Published:** 1898-07

**Authors:** F. R. Collard

**Affiliations:** Wheelock, Texas


### Report of Laparotomy for Obstruction of Bowels.

BY F. R. COLLARD, M. D. WHEELOCK, TEXAS.

[Read before the Brazos Valley Medical Association, at Calvert, Texas, May 10, 1898.]

The report of this case is remarkable from the fact that the patient died; and interesting on account of the nature of the obstruction.

We are too prone, but it is human nature, to publish and boast of our successes, and cover up and hide our failures. We all make mistakes, but it is foolish to make the same mistake twice. We should profit by and publish them, thereby giving others opportunity to be benefited.

The cause of obstruction in the case under consideration was Meckles diverticulum. An abnormality of the small intestine, the remains of the vitellum, or amphalo-mesenteric duct of the embryo, springing from the ilium a short distance above the cæcum, directed forward towards the umbillicus. Sometimes a tube resembling the small intestine and opening on the exterior through the umbillicus; sometimes it extends two or three inches, ending in a fibrous cord which is lost in the umbillicus; or the end may be free and club-shaped, or it may have attachments elsewhere. If it is fixed at both ends a loop of the intestine may slip beneath it and be twisted on itself and become strangulated. It may coil itself into a loop, or if the end is large and free it may knot itself into a snare. In a few cases a loop of the intestine may fall over it, and be so acutely bent, and such traction be made on it as to close the lumen completely. And finally it may allow the intestine to be, as it were, invaginated through its extruding through the umbilicus.*

In this instance the blind end was attached to the mesentery. It was about an inch long and a little larger in diameter than an ordinary lead pencil, somewhat cone shaped, having all the anatomical characteristics of the intestine, and at its open end freely communicating with it, thereby forming a small loop. Into this loop a knuckle of the small intestine had been forced and became strangulated.

The patient was about 30 years of age, of fine physique, family history excellent. Was attacked on Thursday night with symptoms of colic, occasional vomiting; was at the time away from home. Hypodermic injection of half grain of codeine was administered. Friday morning rode (horseback) home (two miles). Still symptoms of colic. Eight grains of calomel given, bowels flooded with stimulating enemas every two or three hours. No action from the bowels; obstruction suspected. Saturday morning one ounce of castor oil given, repeated in the course of the day; still occasional attacks of vomiting, though a major portion of the oil was retained; no action from the bowels; diagnosis of obstruction almost certain; operation advised. I saw the case first on Saturday, and was made aware of the above history and treatment by the attending physician. At this time the pulse was 90, full and regular, temperature a little above normal, and some expression of anxiety; and some tympanites, though not pronounced. I agreed with the diagnosis of obstruction and concurred with the advice of operation.

Friends telegraphed for surgeon 150 miles away; surgeon never arrived until Monday at 5 o'clock p. m. Pulse at this time about 90, full and regular. Preparations made to operate, but decided to wait until Tuesday morning. This I thought bad policy, and think so still. (We are all generals after the battle is fought.)

The city surgeon now took the lead in the case. Tuesday morning came. Local physicians invited in, and Dr. Curry and myself attended. Temperature in open air 40° F.; room heated artificially to 80° F. (Thermometer in the room.) Fine light. Chloroform administered. Hypodermic syringes loaded with strychnine 1-30 gr. and nytroglycerine 1-75 gr., respectively. Chloroform well borne; respiration fine; circulation unchanged.

---

*Moulin's Surgery, pp. 916, 917, third ed., 1895.

Incision in mesial line, beginning two inches above the unbilicus, extending three or four inches below. Peritoneal cavity opened and obstruction soon found. Some recent adhesions had formed but were easily broken up. About half a yard of the intestines had found its way through the loop and was strangulated. This was easily reduced.

Now, the question arises; What should be done with the diverticulum? If permitted to remain there would be danger of obstruction again, but the man had been wearing it thirty years, why not thirty more, or sixty more? It was ligated, double ligature (silk gut) at its point of attachment to the mesentery and severed.

Now another question arises: Should the bowel have been returned and abdominal incision closed at this juncture? Would not the raw cut end attach itself to some other, if not the same site, and perchance form another loop?

It was sévered even with the peritoneal surface of the main gut, after an assistant had squeezed the intestinal contents both ways from the site of attachment and held the now flattened gut between the thumbs and fingers. There was now an aperture full three-quarters of an inch long into the bowel proper. This was closed with Lembert's (?) suture, end doubly guarded by one extra row of uninterrupted stitches. In the meantime yards of the inflated bowel with its contents, the calomel, the oil-was out exposed to the atmosphere, and when the time came for replacing it within, and closing the abdominal incision it was questionable if the bowel could be returned into the abdominal cavity. The surgeon slit a hole into the gut, and its contents began pouring out, and that before there was a receptacle ready to receive them; but one of the attendants caught the greater quantity.

The intestine did not contract on its contents so as to completely empty itself (up to this time the pulse was good and under a hundred). When it was found that the gut would not empty itself unaided it was caught between the thumb and finger and its contents forcibly driven to the slit, from above downwards, and from below upwards. While this procedure was in progress the pulse suddenly and rapidly failed. Nitro-glycerine and strychnine administered hypodermatically. The wound in the bowel was closed as the first had been. The bowel was returned into the abdominal cavity, but not without trouble and delay. The external wound stitched up, and the patient put to bed with a weak, thready pulse of 150°, from which condition he never rallied, and died some eight hours after the operation.

Now, another question arises: If this procedure was necessary, i. e., emptying the bowel through an artificial opening, why was it not emptied through the first opening at the site of the diverticulum? Why make two holes in the bowel when one would have served the purpose? Thus keeping the bowel exposed unnecessarily?

In these days of antiseptic surgery we are too prone to whittle on and expose a man's vitals, and place our sole reliance on our antiseptic treatment; and apparently forget that we are cutting on a patient and not on a cadaver. We depend too much on our ally—antiseptics—and do not keep up our wing of the army. Common sense should teach us that such a course would end in disaster.

While I commend antiseptics both during and after operation, antiseptic treatment, etc., I more highly commend good old horse sense. The truth is, good surgery is good common sense, and applies not only to surgery, but to general practice. In fact, to everything in and out of the profession.

